# Ablation or Resection for Colorectal Liver Metastases? A Systematic Review of the Literature

**DOI:** 10.3389/fonc.2019.01052

**Published:** 2019-10-16

**Authors:** Philipp Kron, Michael Linecker, Robert P. Jones, Giles J. Toogood, Pierre-Alain Clavien, J. P. A. Lodge

**Affiliations:** ^1^Department of HPB and Transplant Surgery, St. James's University Hospital, NHS Trust, Leeds, United Kingdom; ^2^Department of Surgery and Transplantation, Swiss HPB and Transplant Center, University Hospital Zurich, Zurich, Switzerland

**Keywords:** liver surgery, liver resection, colorectal liver metastases, radiofrequency ablation, systematic review

## Abstract

**Background:** Successful use of ablation for small hepatocellular carcinomas (HCC) has led to interest in the role of ablation for colorectal liver metastases (CRLM). However, there remains a lack of clarity about the use of ablation for colorectal liver metastases (CRLM), specifically its efficacy compared with hepatic resection.

**Methods:** A systematic review of the literature on ablation or resection of colorectal liver metastases was performed using MEDLINE, Cochrane Library, and Embase until December 2018. The aim of this study was to summarize the evidence for ablation vs. resection in the treatment of CRLM.

**Results:** This review identified 1,773 studies of which 18 were eligible for inclusion. In the majority of the studies, overall survival (OS) and disease-free survival (DFS) were significantly higher and local recurrence (LR) rates were significantly lower in the resection groups. On subgroup analysis of solitary CRLM, resection was associated with improved OS, DFS, and reduced LR. Three series assessed the outcome of resection vs. ablation for technically resectable CRLM, and showed improved outcome in the resection group. In fact, there were no studies showing a survival advantage of ablation compared to resection in the treatment of CRLM.

**Conclusions:** Resection remains the “gold standard” in the treatment of CRLM and should not be replaced by ablation at present. This review supports the use of ablation only as an adjunct to resection and as a single treatment option when resection is not safely possible.

## Introduction

### Rationale

Colorectal cancer (CRC) is the third most common cancer worldwide (1). At the time of diagnosis, 30–50% of the patients already have (synchronous) or will develop (metachronous) colorectal liver metastases (CRLM) in the further course of their disease (2). In metastatic CRC limited to the liver without extrahepatic disease, resection of liver lesions remains the gold standard with 5 year survivals reported to be over 60% for selected patients (3, 4). The importance of surgery in the treatment of CRLM was recognized very early (5). Richard Cattel performed the first resection of colorectal liver metastases in 1940. However, it took several decades for the impact of liver surgery on overall survival (OS) and disease free survival (DFS) to be recognized (5).

One of the main goals of liver resection for CRLM is to achieve a complete tumor removal with cancer free resection margins (6, 7). With the introduction of better imaging, potent chemotherapy and new surgical approaches, the boundaries of treatment have been expanded in CRLM (6–8). Patients that formerly seemed to be unresectable, nowadays have a chance to undergo potentially curative resection. Even in patients with extensive, bilobar CRLM and an expected marginal future liver remnant (FLR), newly introduced multi-stage resection strategies offer a potential opportunity for cure by allowing time for the liver to regenerate between the stages (6, 9, 10). Existing approaches for multi- stage liver resections are the classical two-stage hepatectomy (TSH) approach and the associating liver partition and portal vein ligation for staged hepatectomy (ALPPS) approach. In the “classical” two stage approach, portal vein ligation (PVL) or portal vein embolization (PVE) is included in the first stage to stimulate liver hypertrophy of the planned FLR, followed by resection in the second step, most usually 4–8 weeks later (after a confirmed appropriate volume increase of the FLR) (11). The other two- stage approach, namely ALPPS, was introduced more recently (10). Besides PVL/PVE, the first step in ALPPS includes transection of the liver parenchyma (12). ALPPS is able to accelerate liver growth facilitating the second step within a shorter period of time, keeping the inter-stage interval short and providing the potential benefit of a higher resection rate compared to the classical two stage hepatectomy approach in extensive colorectal liver disease (8, 13, 14). Despite these developments, extending the limits of resectability, a high percentage of patients with CRLM remain unresectable either due to extensive liver disease or due to comorbidities precluding resection (15). Therefore, a variety of local ablative approaches have evolved to either complement resection or as a single treatment modality for otherwise unresectable CRLM, most commonly radiofrequency ablation (RFA) and microwave ablation (MWA) (16, 17). These local ablative strategies have shown to be safe and feasible in selected patient subpopulations and the approach is well-accepted for patients who are not candidates for resection (18). Over the last years the local ablative strategies have shown promising results, with response rates up to 95% and median survival rates up to 36 months (19).

### Objectives

These encouraging data led to the demand of directly comparing resection and ablation in CRLM to define the roles of the two treatment modalities in the treatment algorithm of CRLM (20).

### Research Question

Since the evidence on this topic is scarce, the aim of this study was, based on a discussion at the EAHPBA 2019 in Amsterdam, the Netherlands, to assess the evidence comparing resection and RFA for the treatment of CRLM.

## Materials and Methods

### Study Design

Since the majority of the available evidence involved RFA (as opposed to other forms of thermal ablation) only, this reviewed considered reports comparing RFA vs. resection in the treatment of CRLM. The study followed the Preferred Reporting Items for Systematic Reviews and Meta-Analysis (PRISMA) statement standards (21). Studies were identified by searching the electronic databases EMBASE, MEDLINE, and Cochrane Library. A professional experienced information specialist from the Main Library of the University of Zurich performed the literature search for published data, between September and December 2018. A combination of subject headings and keywords for liver resection, liver metastases, ablation therapy, cryoablation, thermoablation electrocoagulation, radiofrequency ablation, rectum tumor, colon tumor, and colorectal liver metastases was used for the literature search.

### Search Strategy

#### Keywords for the Search

exp Liver Neoplasms/sc or (exp neoplasm metastases/ or exp neoplasm recurrence, local/) and ((hepatic or liver or hepatocellular or hepato-cellular).mp.) or (hepatic or liver or hepatocellular or hepato-cellular) adj3 (metasta^*^ or secundar^*^ or spread or advanced)).ti,ab. or (hepatic or liver or hepatocellular or hepato-cellular) adj3 ((neoplasm^*^ or cancer^*^ or carcinom^*^ or tumo^*^ or malign^*^) and metasta^*^).ti,ab. or (recurren^*^ adj9 (liver or hepat^*^) adj1 (neoplasm^*^ or cancer^*^ or carcinom^*^ or tumo^*^ or metasta^*^ or malign^*^).ti,ab. exp colorectal neoplasms/ or (colorectal or colon^*^ or rect^*^) adj3 (neoplasm^*^ or cancer^*^ or carcinom^*^ or adenocarcinom^*^ or tumo^*^ or malign^*^).ti,ab. exp Ablation Techniques/ or (ablation or cryotherapy or thermoablati^*^ or “thermo destruc^*^” or “thermal destruc^*^” or “thermocoag^*^” or “thermo coag^*^” or “thermal coag^*^” or electrocoagulation or radiofrequ^*^ or radio-frequ^*^ or rfa or pei or PAI).ti,ab. or (ablati^*^ adj1 (therap^*^ or method^*^ or treatment^*^ or procedure^*^ or surgery or technique^*^).ti,ab. or (injection adj5 (ethanol or “acetic acid”)).ti,ab.

**Figure 1:**
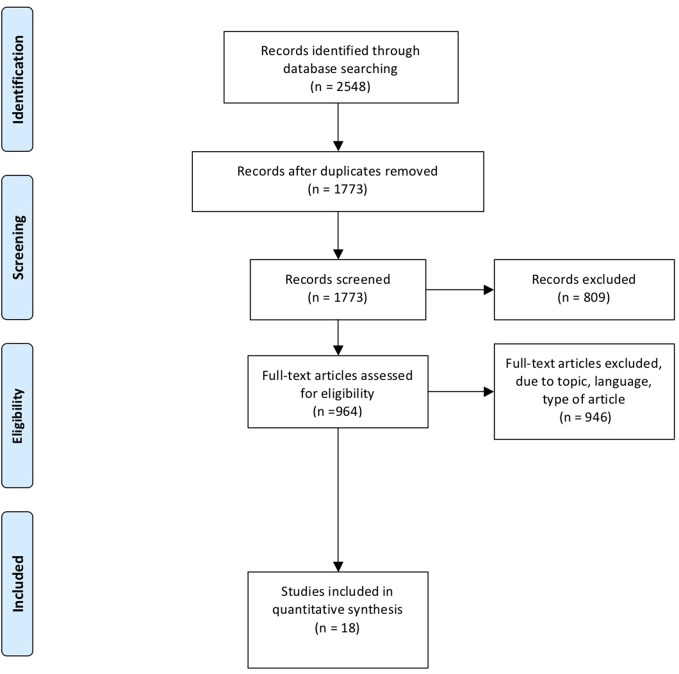
PRISMA flow chart of literature search.

Hepatectomy/or exp Liver Neoplasms/su or (liver or hepat^*^ or surgical) adj3 (resect^*^ or surgery).ti,ab. or (hepatectomy or lobectomy).ti,ab. 1 and 2 and 3 and 4. The PRISMA flow chart is shown in Figure 1. The last electronic literature search was performed on 20 December 2018. PRISMA checklist is shown in Supplementary Figure 1.

### Data Extraction

Two reviewers independently reviewed the abstracts. Suitable abstracts were identified and full text analysis was performed. Discrepancies between reviewers were resolved after discussion between them and the senior authors.

### Data Selection

For final inclusion, studies had to compare the effects of ablation (RFA) and hepatic resection (HR) in the treatment of CRLM. Studies dealing with primary cancer or other ablation strategies than RFA were excluded. Editorials, letters, abstracts, case reports were not included. All included studies had to be available in English.

## Results

### Study Selection and Characteristics

The systematic literature search identified 2,548 records. Excluding duplicates, 1,773 publications were screened according to title and abstract. Eight hundred and nine abstracts were excluded due to language, topic, tumor entity, type of manuscript, or because the full text was not available. In total, 964 publications were eligible for full-text review. A further 946 were excluded due to language, topic or tumor type. A total of 18 studies were therefore included in the final analysis (Figure 1) (22–38).

### Non-randomized Studies and Patients Demographics

Eighteen non-randomized studies were identified. Seventeen (94%) of the eighteen studies included were retrospective studies, and only one was prospective. Among the 18 studies, seven (39%) compared the outcomes following RFA vs. resection in solitary CRLM (23, 24, 26, 28, 34, 37, 38). Three of the 18 studies (17%) compared the two treatment modalities in potentially resectable CRLM. In total, 2,667 patients were treated either with RFA (*n* = 998, 37%) or hepatic resection (*n* = 1669, 63%). In 11.1% of the studies included RFA was performed by surgeons. In 33.3% of the cases RFA was performed by radiologists. In 55.6% of the manuscripts included it was not clearly stated who performed the intervention. Gender was reported for 2,420 patients, with 1,505 males (62.2%) and 915 (37.8%) females. The patient study and detailed characteristics of the interventions are summarized in Table 1. The study cohorts except one manuscript were not matched. RFA was in the majority of the studies performed when surgical resection was not an option due to insufficient future liver remnant, unresectable disease or underlying patient comorbidities precluding surgery. In one study (6%) RFA was the first line treatment offered to all patients (35). Ko et al. performed RFA in all patients declining HR as a first line treatment (38). Wang et al. performed RFA if a complete necrosis based on tumor size and position could be achieved, patients comorbidities that precluded general anesthesia or surgery, and relying on patient choice (40). In this analysis the cohorts were matched according to the propensity score (40).

**Table 1:** Identified studies for the systematic review according to PRISMA guidelines.

**References**	**Study**	**Modality**	**Number of patients**	**Gender m/f**	**RFA via US/CT**	**Open/percutaneous**
Abdalla et al. (22)	Retrospective	RFA	57	Unclear	US	All RFA open during
		Resection	190	Unclear		Laparotomy
Agcaoglu et al. (25)	Retrospective	RFA	295	196/99	US	All RFA done during
		Resection	94	50/44		Laparoscopy
Aliyev et al. (26)	Retrospective	RFA	44	24/20	US	All RFA done during
		Resetion	60	34/26		Laparoscopy
Aloia et al. (23)	Retrospective	RFA	30	23/7	US	Percutaneous/intraop
		Resection	150	85/65		
Gleisner et al. (27)	Retrospective	RFA	11	7/4	US	All RFA open during
		Resection	192	121/71		Laparotomy
Hur et al. (28)	Retrospective	RFA	25	15/10	US	Intraop/percutaneous
		Resection	42	27/15		
Kim et al. (36)	Retrospective	RFA	177	121/56	US	Intraop/percutaneous
		Resetion	278	168/110		
Kim et al. (29)	Retrospective	RFA	17	12/5	US	Intraop/percutaneous
		Resection	43	28/15		
Ko et al. (38)	Retrospective	RFA	17	9/8	Modality unclear	Intraop/percutaneous
		Resection	12	9/3		
Lee et al. (30)	Retrospective	RFA	37	26/11	US	Percutaneous
		Resection	116	76/40		
Lee et al. (37)	Retrospective	RFA	28	23/5	US	Percutaneous/intraop
		Resection	25	14/11		
McKay et al. (31)	Retrospective	RFA	43	25/18	US	All RFA open during
		Resection	58	29/29		Laparotomy
Oshowo et al. (24)	Retrospective	RFA	25	11/14	US/CT/MRI	Percutaneous
		Resection	20	10/10		
Otto et al. (35)	Prospective	RFA	28	20/8	CT	Percutaneous
		Resection	82	49/33		
Park et al. (32)	Retrospective	RFA	30	22/8	US/CT	Percutaneous
		Resection	59	41/18		
Reuter et al. (33)	Retrospective	RFA	66	46/20	US	Intraop
		Resection	126	69/57		
Wang et al. (39)	Retrospective	RFA	46	29/17	US	Unclear
		Resection	92	58/34		
White et al. (34)	Retrospective	RFA	22	8/14	CT	Unclear
		Resection	30	20/10		

*RFA, Radiofrequency ablation; ns, not significant; DFS, disease free survival; OS, overall survival; y, year; PFS, Progression free survival; US, Ultrasound*.

### Overall Survival (OS)

Of the 18 studies included, eight (44%) studies showed a significantly better overall survival in patients treated with resection compared to RFA (22, 23, 25, 27, 28, 30–32; Table 2). Ten studies (56%) did not show any difference in terms of overall survival in favor of either treatment modality.

**Table 2:** DFS, OS, and Local recurrence rate of the studies included.

**References**	**Modality**	**DFS in months**		**Median OS in months**		**5y OS in %**		**Local recurrence rate in %**	
Abdalla et al. (22)	RFA	**7**	**Significant**	**25**	**Significant**	**21**	**Significant**	**44**	**Significant**
	Resection	**31**		**72**		**58**		**11**	
Agcaoglu et al. (25)	RFA	**8**	**Significant**	**31**	**Significant**	**17**	**Significant**	69	ns
	Resection	**21**		**60**		**58**		40	
Aliyev et al. (26)	RFA	Not reported		22	ns	47	ns	**18**	**Significant**
	Resection	Not reported		25		57		**4**	
Aloia et al. (23)	RFA	**0**	**Significant**	**47**	**Significant**	**27**	**Significant**	**37**	**Significant**
	Resection	**50**		**126**		**71**		**5**	
Gleisner et al. (27)	RFA	**0**	**Significant**	**38.1**	**Significant**	**28.3**	**Significant**	**41.3**	**Significant**
	Resection	**41.3**		**73.4**		**57.4**		**2**	
Hur et al. (28)	RFA	Not reported		not reported		**25.5**	**Significant**	28	ns
	Resection	Not reported		not reported		**50.1**		9.5	
Kim et al. (36)	RFA	Not reported	**Significant**	not reported		14.3	ns	Not reported	
	Resection	not reported		not reported		34.6		Not reported	
Kim et al. (29)	RFA	26.9	ns	30	ns	47.1	ns	76.5	ns
	Resection	35	3 y DFS in %	57		53.3	3y OS	60.2	
Ko et al. (38)	RFA	17.6	ns	na		37.8	ns	Not reported	
	Resection	22.2		na		66.7		Not reported	
Lee et al. (30)	RFA	21.1	ns	40	ns	48.5	ns	**29.7**	**Significant**
	Resection	23.7		44.7		65.7		**6.9**	
Lee et al. (37)	RFA	**10**	**Significant**	**24**	**Significant**	Not reported		**42.9**	**Significant**
	Resection	**24**		**41**		Not reported		**8**	**Recurrence at margin**
McKay et al. (31)	RFA	15	ns	**30**	**Significant**	**23**	**Significant**	**60**	**Significant**
	Resection	17		**44**		**43**		**7**	
Oshowo et al. (24)	RFA	Not reported		34	ns	52.6	ns	Not reported	
	Resection	Not reported		41		55.5	3 y OS	Not reported	
Otto et al. (35)	RFA	Not reported		45	ns	60	ns	**32**	**Significant**
	Resection	Not reported		56		67	3 y OS	**4**	
Park et al. (32)	RFA	**Not reported**	**Significant**	**36**	**Significant**	Not reported	**Significant**	**23.3**	**Significant**
	Resection	**Not reported**		**56**		Not reported		**1.7**	**Recurrence at margin**
Reuter et al. (33)	RFA	Not reported		27	ns	21	ns	**17**	**Significant**
	Resection	Not reported		36.4		23		**2**	
Wang et al. (39)	RFA	**14**	**Significant**	74	ns	71.7	ns	15.2	ns
	Resection	**22**		59		66.8	3 y OS	6.5	
White et al. (34)	RFA	**17**	**Significant**	31	Not clearly stated	Not reported		55	Not reported
	Resection	**68**	**PFS**	80		Not reported		12	Local disease progression

*RFA, Radiofrequency ablation; ns, not significant; DFS, disease free survival; OS, overall survival; y, year; PFS, Progression free survival. The bold represent significant values*.

### Disease Free Survival (DFS)/Progression Free Survival (PFS)

Eight (44%) studies showed a significantly longer DFS for patients undergoing hepatic resection compared to RFA. One study (6%) showed a significant better progression free survival (PFS) in the resection group (Table 2). Half of the studies showed a significantly better DFS/PFS benefit for resection (Table 2). In four (22%) of the 18 studies included, the DFS or the significance between the two treatment groups are not reported. Almost a third (28%) of the studies did not show any significance in favor of one group (Table 2).

### Local Recurrence Rate

In 10 (56%) of the 18 included studies the local recurrence rate was significantly lower in patients treated with hepatic resection compared to RFA. Three (16%) studies included did not report their local recurrence rate following treatment with RFA or hepatic resection. Five (28%) studies did not show any significant differences in terms of local recurrence rate following the two treatment modalities in favor of either group. Details are shown in Table 2.

### Subgroup Analysis

#### Solitary Colorectal Liver Metastases

There were seven studies which assessed outcomes in patients with only a solitary liver metastases (23, 24, 26, 28, 34, 37, 38). In total 630 patients were treated for solitary CRLM. Sixty eight percent (*n* = 430) of patients were treated with resection and 32% (*n* = 200) of patients were treated with ablation. Of the patients treated with ablation, 116 (58%) were male and 84 (42%) female. In the resection group 61% (*n* = 261) were male and 39% (*n* = 169) female. Of the seven studies, only one (14.3%) showed a significant advantage for resection in terms of 5 year DFS (50 vs. 0%; *p* = 0.001). White et al. demonstrated a significantly longer PFS in patients undergoing resection (17 vs. 68 months, *p* < 0.01). Two studies (28.6%) showed a significantly higher 5 year overall survival in the hepatic resection (HR) group (Aloia et al. 27 vs. 71%; *p* < 0.001; Hur et al. 26 vs. 50%, *p* = 0.026) (23, 28); Ko et al. showed a higher DFS and 5 year OS in the HR group, but these differences were not significant (5 year DFS 18 vs. 22%; 5 year OS 38 vs. 67%) (38). Three studies (43%) showed a significantly higher local recurrence rate in the RFA group [Table 3; (23, 26, 37)]. Two additional studies also showed a significant advantage for HR in terms of 5 year recurrence free survival/PFS over RFA in the treatment of solitary colorectal liver metastasis (28, 34, 37). In total, three studies (43%) showed a significant advantage for HR compared to RFA on recurrence.

**Table 3:** DFS, OS, and Local recurrence rate of the studies included with solitary CRLM.

**References**	**Modality**	**DFS in months/5 y DFS in %**		**Median OS in months**		**5 y OS in %**		**Local recurrence rate in %**	
Aliyev et al. (26)	RFA	Not reported	ns	22	ns	47	ns	**18**	**Significant**
	Resection	Not reported		25		57		**4**	
Aloia et al. (23)	RFA	**0**	**Significant**	**47**	**Significant**	**27**	**Significant**	**37**	**Significant**
	Resection	**50**		**126**		**71**		**5**	
Hur et al. (28)	RFA	Not reported		Not reported		**25.5**	**Significant**	28	ns
	Resection	Not reported		Not reported		**50.1**		9.5	
Ko et al. (38)	RFA	17.6	ns	Not reported		37.8	ns	na	
	Resection	22.2		Not reported		66.7		na	
Lee et al. (37)	RFA	21.1	ns	40	ns	48.5	ns	**29.7**	**Significant**
	Resection	23.7		44.7		65.7		**6.9**	
Oshowo et al. (24)	RFA	Not reported		34	ns	52.6	ns	Not reported	
	Resection	Not reported		41		55.5	3 y OS	Not reported	
White et al. (34)	RFA	**17**	**Significant**	**31**	**Significant**	Not reported		55	Not reported
	Resection	**68**	**PFS**	**80**		Not reported		12	Local disease progression

*RFA, Radiofrequency ablation; ns, not significant; DFS, disease free survival; OS, overall survival; y, year; PFS, Progression free survival. The bold represent significant values*.

In a subgroup analysis, four out of these seven studies (57%) provided enough data to assess the effects of RFA vs. HR for solitary CRLM lesions ≤3 cm (23, 26, 28, 37, 38). A total of 255 patients were included, 125 (49%) underwent RFA and 130 (51%) patients underwent resection for solitary CRLM ≤3 cm in size. Aloia et al. demonstrated a significantly higher 5 year OS in the HR group (71 vs. 18%; *p* = 0.006; Table 4) and a significantly lower local recurrence rate for the resection group in this subgroup analysis (3 vs. 31%; *p* < 0.001) (23). Aliyev et al. showed a significant advantage for resection compared to RFA in terms of local recurrence (4 vs.18%; *p* = 0.012) (26, 34). Hur et al. failed to show any significance for one of the treatment groups when analyzing this particular subpopulation but did show a significant higher survival rate (42 vs. 30%; *p* < 0.001) as well as a significant longer LRFS (81 vs. 50 months; *p* = 0.013) in the group of solitary CRLM > 3 cm in the resection group (28, 38). These observations are further supported by the results from Ko et al. (38). This group showed a significant lower 5 year OS as well as a significantly lower 5 year DFS in the RFA group for patients with solitary CRLM > 3 cm (5 year OS: 0 vs. 57%; *p* = 0.005 5 year DFS: 0 vs. 38.1%; *p* = 0.013).

**Table 4:** DFS, OS, and Local recurrence rate of the studies included with solitary CRLM ≤ 3cm.

**References**	**Modality**	**DFS in months**	**Median OS in months**		**5 y OS in %**		**Local recurrence rate in %**	
Aliyev et al. (26)	RFA	Not reported	22	ns	47	ns	**18**	**Significant**
	Resection	Not reported	25		57		**4**	
Aloia et al. (23)	RFA	Not reported	Not reported		**18**	**Significant**	**31**	**Significant**
	Resection	Not reported	Not reported		**71**		**3**	
Hur et al. (28)	RFA	Not reported	Not reported		55.4	ns	13.3	ns
	Resection	Not reported	Not reported		56.1		4.3	
Ko et al. (38)	RFA	Not reported	Not reported		80	ns	Not reported	
	Resection	Not reported	Not reported		49.5		Not reported	

*RFA, Radiofrequency ablation; ns, not significant; DFS, disease free survival; OS, overall survival; y, year. The bold represent significant values*.

#### RFA vs. Resection for resectable CRLM

In three of the 18 studies (16.7%) hepatic resection was compared to RFA in potentially resectable CRLM (35, 38, 39). Two of the studies were retrospective studies, with one prospective series. In total 277 patients were included in this subgroup analysis. One hundred and eighty-six patients (67%) underwent resection and 91 patients (33%) ablation. In the resection group 116 (63%) were male and 70 (38%) female. Sixty four percent of patients undergoing RFA were male and thirty six percent female. In the study by Otto et al. RFA was considered as first line treatment. Surgery was only performed in patients not suitable for RFA due to number, size, or location of the metastases (35). Ko et al. only performed RFA for CRLM when patients refused HR after being informed about the two different treatment options, potential complications and survival rates (38). In the manuscript by Wang et al. indications for RFA were as follows: complete necrosis of the CRLM feasible, tumor size and position, comorbidities precluding HR and patient choice (39).

Wang et al. and Otto et al. demonstrated a significant benefit of resection in terms of local recurrence rate and time to local recurrence compared to RFA in resectable CRLM (35, 39). The local recurrence rate in in the resection group was 4 vs. 32%; *p* < 0.001 in the RFA group (35). Wang et al. showed a significant higher intrahepatic recurrence (37 vs. 12%; *p* = 0.001*)* in the RFA group as well as a significant shorter time to recurrence in the RFA group. Furthermore, they demonstrated a significant longer survival in the resection group (22 vs. 14 months; *p* = 0.032; Table 5) (39).

**Table 5:** DFS, OS, and Local recurrence rate of the studies included with technically resectable CRLM.

**References**	**Modality**	**DFS in months/5 y DFS in %**		**Median OS in months**		**5 y OS in %**		**Local recurrence rate in %**	
Ko et al. (38)	RFA	17.6	ns	Not reported		37.8	ns	Not reported	
	Resection	22.2		Not reported		66.7		Not reported	
Otto et al. (35)	RFA	not reported		Not reported		67	ns	**32**	**Significant**
	Resection	not reported		Not reported		60	3 y OS	**4**	
Wang et al. (39)	RFA	**14**	**Significant**	74	ns	71.7	ns	15.2	ns
	Resection	**22**		59		66.8	3 y OS	6.5	

*RFA, Radiofrequency ablation; ns, not significant; DFS, disease free survival; OS, overall survival; y, year. The bold represent significant values*.

## Discussion

Hepatic resection is the treatment of choice for colorectal liver metastases with 5 year OS approaching 60% (40, 41). Ablation techniques have shown great promise in other disease types but this does not mean that it should necessarily be applied to CRLM (16, 42). Indeed, there remains a lack of clarity surrounding the precise role of ablation compared to surgery for CRLM. The American Society of Clinical Oncology (ASCO) guidelines have highlighted the wide variation in overall survival and local recurrence rates after ablation, and suggested that in the absence of adequate data resection should remain the gold standard treatment for resectable disease (43). Despite these concerns, ablation may still have a role as an adjunct to resection. Patients with small volume resectable metastases who are not sufficiently fit to undergo liver resection can be considered for ablation as should those with limited liver metastases who have insufficient liver volume to undergo resection due to tumor position (24, 44).

Based on a discussion at the EAHPBA 2019 in Amsterdam, the Netherlands, this systematic review was conducted to investigate the contemporary evidence on ablation vs. resection for the treatment of CRLM. So far there are no completed randomized studies on this topic. The COLLISION trial is a randomized controlled trial (RCT) comparing the efficacy of ablation and resection in patients with at least one resectable and ablatable CRLM with a maximal diameter of not more than 3 cm (45). The protocol of this trial was published in 2018 but results are still pending. Another ongoing RCT is the HELARC trial (Trial ID NCT02886104). This study aims to compare surgical and ablative strategies for the treatment of resectable synchronous CRLM, with patients randomized to resection of the primary tumor and resection or ablation of the liver metastases. The study has an estimated completion date of 2026. A major UK trial (LAVA) comparing the impact of resection and ablation on disease free survival was aimed at patients mainly selected on grounds of age and comorbidities and this recently closed due to failure to recruit adequate patient numbers (46). One interesting, unpublished study on this topic was presented at the EAHPBA 2019, by Engstrand et al. comparing the results of MWA and resection in CRLM in a propensity score matched cohort with promising results for MWA. Due to the limited evidence of published data on MWA and other interventional ablation approaches in direct comparison with resection, RFA only was chosen as the primary treatment of choice for comparison.

### Summary of Main Findings

The present systematic review has shown a superior oncological outcome following hepatic resection in comparison to RFA in the treatment of CRLM. This systematic review has identified a higher overall- and DFS in the HR group. RFA as a single treatment was associated with a significantly higher local recurrence. Even in a subgroup analysis of solitary CRLM and solitary CRLM ≤ 3 cm HR showed oncological benefits in DFS, OS and local recurrence rate. Within a subgroup analysis comparing the effects of HR and RFA in technically resectable CRLM, RFA produced inferior results.

Currently there are no results from RCTs available, directly comparing the outcome of the two treatment modalities. There are RCTs registered and protocols of these trials published and their results are eagerly awaited. The failure of the UK LAVA trial to recruit patients also highlights a potential equipoise amongst surgeons regarding ablation vs. resection for resectable disease. Although screening data has yet to be released for this study, it may also be that patients were unwilling to be randomized to ablation when surgery was an option. These data will be vital to assess the feasibility of future trials.

RFA gained popularity as an interventional treatment option for small hepatocellular carcinomas and on this background of relative success RFA was postulated to be an effective alternative also in CRLM, especially in the treatment of CRLM ≤ 3 cm (42, 47–50). However, in the absence of evidence, this was a dangerous assumption. RFA is attractive in that it offers a minimally invasive treatment alternative compared to HR, in conjunction with lower post-interventional morbidity, lower complication rates and shorter hospital stays compared to resection (22, 51–53). In a review from Weng et al. overall complication rates following RFA were significantly lower compared to hepatic resection (3.9 vs. 18.3%; *p* < 0.01). Van Amerong et al. support these results in a meta-analysis published in 2017 (48). But these short- term advantages do not translate into an oncological benefit. This systematic review identified a higher OS in the resection group compared to RFA. Eight (44%) of the 18 non-randomized studies included in this analysis showed a significantly higher OS following resection compared to RFA (22, 23, 25, 27, 28, 30–32). These results are similar to the results of other meta-analyses (48, 49).

A potential explanation of the lack of OS benefit in five of the studies could be that the investigators were comparing the effects of the two treatment modalities for solitary CRLM with better long-term outcomes (24, 26, 34, 37, 38). Indeed, it could be argued that these data suggest that patients with solitary metastases do equally well whether treated with ablation or resection. This lack of difference in long term survival is made even more intriguing by the results of Aliyev et al. who showed that the local recurrence rate was significantly lower in patients undergoing resection compared to ablation (26). It may therefore be that local lesional recurrence in biologically good prognosis patients has no impact on long term outcome. One additional study also showed a significant advantage for HR in terms of local recurrence free survival over RFA in the treatment of solitary CRLM (34, 37). White et al. showed a lower disease progression rate in the HR group (12 vs. 55%) that was not significant in their analysis (34). Ko et al. showed a trend toward an improved DFS and 5 year OS in the HR group, but these differences were not significant, potentially triggered by a very limited number of patients treated: 13 patients were assigned to the RFA group and only five patients were included in the resection cohort (38). When looking a little bit closer at the subgroup analysis of this study, Ko et al. did show a significant advantage for resection compared to RFA for bith DFS and 5 year OS for CRLM > 3 cm despite the very limited number of patients included in this retrospective analysis. This finding is confirmed by other studies and a tumor size > 3 cm was identified as an independent negative predictor of outcome following RFA treatment (50).

Since Interventional treatment modalities have gained popularity in the treatment of small tumors, RFA has been postulated to be an effective treatment alternative, especially in the treatment of CRLM ≤ 3 cm (42, 47–50). However, this systematic review has shown in a subgroup analysis that HR is still superior compared to RFA even for solitary CRLM ≤ 3 cm. Aloia et al. also showed a significant advantage of HR compared to RFA in this subgroup analysis in terms of LR rate (3 vs. 31%; *p* < 0.001), 5 year LRFS (97 vs. 66%; *p* < 0.001), and 5 year OS (72 vs. 18%; *p* = 0.006). Other studies have also demonstrated a significant impact for HR on local control in this particular subgroup (26, 34). One potential explanation of the missing significance in LR rate, LR free survival, DFS 5 year and 5 year overall survival in the HR group in the two other studies might be that these studies were underpowered. Hur et al. included 38 patients in total (15 RFA and 23 HR) and 18 patients (13 RFA and 5 HR) were included in the study conducted by Ko et al., respectively.

The difference in success rate for RFA for CRLM ≤ 3 cm compared to the initial very promising treatment outcomes in HCC might be explained by a different biological behavior among the two tumor entities. There is existing evidence that CRLM are assessed as an independent risk factor, negatively influencing the outcome following treatment. These findings of superiority of HR in the treatment of CRLM, even in metastases ≤ 3 cm, are supported by other studies as well, although some groups claim that tumor size ≤ 3 cm and solitary metastases are prognostic characteristics favorable to RFA (23, 48, 49).

Outcome is not only predicted by tumor size alone. The approach of doing RFA itself has a significant impact on the outcome. There is no consensus among experts which treatment approach is most favorable: percutaneous, laparoscopic, or open (laparotomy). Existing evidence suggests that open RFA is associated with a significantly lower risk of LR compared to percutaneous treatment (54–56). In a meta- analysis done by Mulier et al., the percutaneous approach was identified as an independent risk factor for poor outcome, independent of tumor size. The potential short term advantages of the percutaneous approach in terms of reduced invasiveness and reduced morbidity do not counterbalance the long-term effects of inferior oncological outcome of this approach (54). When trying to assess the efficacy of laparoscopic RFA in comparison to open RFA, results analyzing the outcome following these two treatment modalities are much harder to find. In a manuscript published by Mulier et al. the authors showed in their analysis that the local recurrence rate following laparoscopic RFA was higher compared to open RFA even for solitary metastases ≤3 cm (26.3 vs. 1.7%), respectively (57). However, this paper was published in 2008. Improvements in imaging technologies may mean that percutaneous ablation is now better able to accurately target and destroy lesions. It will however, for the moment at least, rely on operator dependability, whatever approach is used. Furthermore, in 11% of the studies included, RFA was performed by surgeons and in 33.3% by radiologists. In 55.6% it is not clearly stated who performed the intervention. If the ablation procedure was not performed by experienced radiologists this might potentially be an additional factor explaining the superior outcome of surgery.

Recent studies have shown that intraoperative ultrasound is associated with a higher accuracy in detecting CRLM compared to the percutaneous treatment approach (58). The last argument in favor for surgical RFA may be the better access. The surgical approach, especially the open approach, gives the surgeon a maximal freedom of placing the electrodes to treat the hepatic disease whereas the percutaneous approach only offers a very limited window of access (59, 60). This advantage of better accessing the metastases in open RFA might also explain the advantage in treatment efficacy compared to the laparoscopic RFA approach.

The thermal ablation of colorectal metastases has been clearly demonstrated to result in complete tumor destruction in experimental models (61). Any difference in long- term outcomes seen between ablation and resection must therefore be explained by a different reason. This may be because occult micrometastases are removed during hepatic resection surgery. Although there is parity for OS, parenchymal sparing resection is also associated with more local recurrence but not inferior in OS due to more repeat resections in order to achieve the OS. This may be the same for RFA (62). Lesional recurrence rates varied in this review, and may explain some of these long- term differences. However, the EORTC 40004 study of RFA for irresectable CRLM reported a lesional recurrence rate of 10% and so likely better reflects contemporary management. More importantly, selection for ablation is likely to represent a biologically worse cohort. The failure of trials of ablation and resection to recruit suggest that surgeons will try and offer surgical resection unless the oncological prognosis is dismal, or patients are unfit for surgery. It may therefore be selection for ablation itself reflects a worse prognosis disease.

Ablation does offer some advantages, including lower post-interventional morbidity, lower complication rates and shorter hospital stays compared to resection but these advantages do not compensate the inferior oncological outcome of RFA compared to resection (22, 51–53). The mentioned short- term benefits of RFA are more a justification for RFA as a treatment tool for selected patient subgroups.

Thus, the available evidence shows that hepatic resection is superior compared to RFA in the treatment of CRLM. In fact, according to the available evidence and results, it is hard to justify a RCT comparing RFA and HR in the treatment of resectable CRLM even in solitary CRLM ≤3 cm since no study, especially in this subgroup analysis could show an advantage for RFA.

### Limitations

The clear strength of this systematic review is, that it provides a comprehensive picture of all available evidence on RFA vs. resection in the treatment of CRLM. As no RCTs are available on this topic, the results of the included pro-/retrospective studies have to be interpreted with caution, particularly as the majority of the studies reviewed were retrospective. We found that the groups compared in the different studies were inhomogeneous in terms of patient characteristics. In the majority of the studies included, patients assigned to RFA were not suitable for resection due to medical fitness or resectability: patients eligible for RFA seemed to be unresectable or too frail for resection, clearly illustrating a selection bias representing a particular subgroup of patients with poor prognosis in the RFA cohorts. Therefore, the level of available evidence is low. In addition, the number of patients included in several studies was limited. These studies might be underpowered to detect any potential significance in the outcomes.

### Outlook

So far there is only limited evidence available in matched cohorts comparing the outcome of these two treatment modalities. The results of the HELARC trial as well as the study results from Engstrand et al. in their propensity score matched cohort are eagerly anticipated. Furthermore, with the introduction of new technologies e.g., 3D navigation, multi needle ablation and robotic approaches RFA has further improved. There are promising results showing the efficacy of stereotactic radiofrequency ablation (SRFA) even in the treatment of CRLM up to 13 cm (63). But these new treatment approaches need further testing in direct comparison with HR.

## Conclusions

So far based on the available evidence resection remains the gold standard in the treatment of CRLM, and cannot be replaced by ablation at present. Although interventional treatment approaches have gained popularity in other tumor entities with promising results in certain subgroups, the available data in this systematic review does not support the use of RFA as a solitary curative treatment in CRLM. We recognize, however, that in the treatment algorithm for CRLM ablation has a role as an adjunct to surgery or as a single treatment option in selected patient subgroups, especially in the treatment of multimorbid patients.

## Data Availability Statement

All relevant data is contained within the manuscript. All datasets for this study are included in the manuscript and the Supplementary Files.

## Author Contributions

All authors: conception and design, manuscript writing, and final approval of manuscript. PK and ML: provision of study material or patients, collection and assembly of data, and data analysis and interpretation.

### Conflict of Interest

The authors declare that the research was conducted in the absence of any commercial or financial relationships that could be construed as a potential conflict of interest.
